# Prenatal diagnosis of cri-du-chat syndrome by SNP array: report of twelve cases and review of the literature

**DOI:** 10.1186/s13039-019-0462-0

**Published:** 2019-12-09

**Authors:** Jiasun Su, Huayu Fu, Bobo Xie, Weiliang Lu, Wei Li, Yuan Wei, Qiang Zhang, Shengkai Wei, Qiuli Chen, Yingchi Lu, Tingting Jiang, Jingsi Luo, Zailong Qin

**Affiliations:** 1 Department of Genetic and Metabolic Central Laboratory, Guangxi Maternal and Child Health Hospital, Guangxi Birth Defects Prevention and Control Institute, No.59, Xiangzhu Road, Nanning, China; 2 Department of Genetic Counseling, Guangxi Maternal and Child Health Hospital, No.225, Xinyang Road, Nanning, China

**Keywords:** Cri-du-chat syndrome, Prenatal diagnosis, SNP microarray, Ultrasound findings

## Abstract

**Background:**

Cri-du-chat syndrome (CdCS; OMIM#123450) is a classic contiguous gene syndrome caused by chromosome 5p terminal deletion (5p-), which characterized by a high-pitched cat-like cry, developmental delay, severe psychomotor, mental retardation, and dysmorphic features in infancy. Prenatal diagnosis of CdCS is difficult due to the non-specific ultrasound features. And reports using array analysis are rare. This study presented the first retrospective analysis of prenatal series of CdCS fetuses diagnosed by single nucleotide polymorphism (SNP) array in China.

**Case presentation:**

A total of 35,233 pregnant women were enrolled from Jan 2014 to April 2019 in our center, there are twelve 5p- cases with abnormal sonographic signs revealed by SNP array, giving an incidence of 0.034% (12/35,233). Clinical information and molecular basis included: maternal demographics, indications for invasive testing, sonographic findings and SNP array results. Among all the 5p- cases revealed, nine cases were diagnosed by both karyotyping and SNP array, three cases were detected only by SNP array. Half of our cases (6/12) had an isolated 5p terminal deletion, which sizes ranged from 9.0 Mb to 30 Mb. The other half of cases (6/12) characterized by unbalanced translocation, with sex ratio 7:5 (female: male), when combine the clinical features observed from this study and available literature, the most frequent anomaly observed in prenatal ultrasound examination of CdCS was cerebral abnormalities, accounted for 44.4% (16/36) of the existing cases. Features that are less consistent included: choroid plexus cyst (13.8%, 5/36), single umbilical artery (13.3%, 4/30), ventricular septal defect (11.1%, 4/36), hydrops fetalis (8.3%, 3/36), ascites (8.3%, 3/36), increased NT/NF (8.3%, 3/36), absent/severely hypoplastic nasal bone (5.5%, 2/36), in order.

**Conclusion:**

Prenatal findings such as cerebral abnormalities, absent/hypoplastic nasal bone, hydrops fetalis, ascites or encephalocele may act as suggestive signs of CdCS or other microdeletion/duplication syndromes. Combining typical karyotyping with chromosomal microarray analysis (CMA) is a definitive method for a precise diagnosis of CdCS and provides more accurate results in order to offer genetic counseling to families which need to deal with cryptic aberrations.

## Background

Cri-du-chat syndrome (CdCS; OMIM#123450) is a well-known contiguous gene syndrome caused by chromosome 5p terminal deletion, first reported by Lejeune et al. in 1963 [1]. The prevalence of CdCS in postnatal was reported as 1 in 15,000 to 1 in 50,000 worldwide and the female to male ratio was estimated approximately 1.3:1 [2, 3]. Clinical features of CdCS patients in postnatal were characterized a high-pitched cat-like cry, developmental delay, severe psychomotor and mental retardation, dysmorphic features including microcephaly, hypertelorism, epicanthal folds, broad nasal bridge, short neck and micrognathia, other structural anomaly may include limbs abnormalities, scoliosis, brain hypoplasia or agenesis, cardiovascular defect, renal abnormalities and genitourinary anomalies [4, 5].

Most of the CdCS cases were caused by a de novo 5p interstitial or terminal deletion, which sizes ranged from 560 kb to 40 Mb [6, 7]. In fact, approximately 80–90% of these cases were paternal in origin and 10–15% were resulted from an unbalanced parental translocations [4]. Other rare complex conditions include 5p-mosaicism (1.4%), inversions (0.5%), and ring chromosomes (0.5%) [2, 8].

Several critical regions contributed the specific phenotype of CdCS had been proposed, however, no common recurring breakpoint have been identified on the correlation between deletion size and severity of clinical features. In the terminal 5p region, a variety of genes have been suspicious to be the cause of this disorder [2] .

In current publications, no specific prenatal ultrasound signs related with CdCS had been proposed since few reports of prenatal diagnosis of CdCS had been published. Presentations such as increased nuchal translucency (NT), prominent glabella, micrognathia, short philtrum, cleft lip/palate, cystic cerebral lesions, abnormal nasal bone or renal hypoplasia and isolated ascites were previously reported in cases of CdCS [9–22]. As CMA is being widely utilized in both postnatal and prenatal diagnosis, the specific prenatal ultrasound signs of CdCS could probably be refined. Herein, we reported the first retrospective analysis of prenatal series of CdCS fetuses by presenting 12 prenatal 5p- cases detected by SNP-microarray in China, we evaluated the deletion coordinates through reviewing the literature and attempted to define the fetal sonographic features for warranting diagnosis of CdCS.

## Case presentation

### Subjects

Between January 2014 and April 2019, A total of 35,233 pregnant women were referred to our center to perform invasive prenatal diagnosis for abnormalities based on fetal ultrasound anatomy scans or maternal serum screening. Fetal ultrasound anatomy scans were routinely performed for pregnant women by senior sonographers using GE E8 ultrasound machines (General Electric Healthcare, US). The indication included: fetus with ultrasound abnormalities (intrauterine growth restriction, increased NT thickness, cleft lip/palate, cystic cerebral lesions, abnormal nasal bone or renal hypoplasia, etc.), positive maternal serum screening test or NIPT high risk for aneuploidy, etc. This was a retrospective study and approved by the Medical Ethics Committee of the Guangxi Maternal and Child Health Hospital and written consent from the parents.

### Molecular and cytogenetic detection

Chorionic villi sampling, amniocentesis or cordocentesis was performed under ultrasound guidance after informed consent. Genomic DNA was extracted using QIAamp DNA Blood Mini Kit (Qiagen, Germany) according to the manufacturer’s protocol. Single-nucleotide polymorphism (SNP) microarray testing was performed using Illumina HumanCytoSNP-12 v2.1 BeadChip (Illumina, USA) and the copy number variation positions were shown according to the human Feb. 2009 (GRCh37/hg19) assembly. The laboratory policy at the time of testing was not to report well established polymorphisms, CNVs that do not contain genes and categorized as benign or likely benign base on the ACMG guideline [23]. Karyotyping was performed for the available samples according to the standard procedure as described previously [24].

## Results

**Fig. 1:**
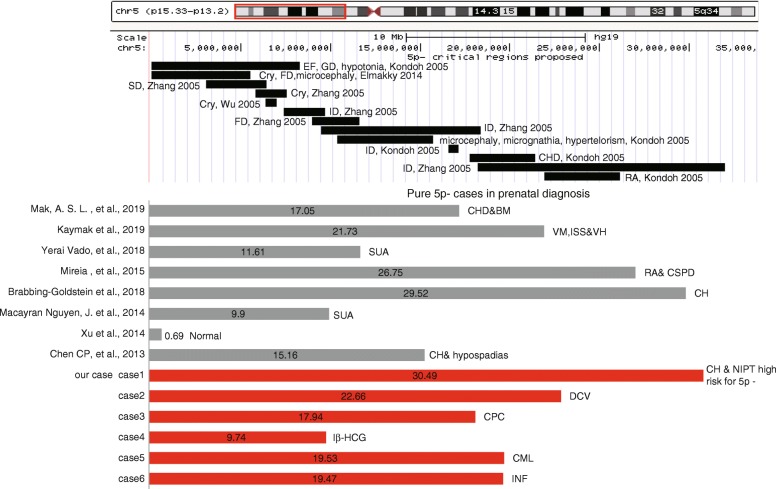
The schematic presentation of the ultrasound findings of isolated 5p deletion cases in prenatal diagnosis, detailed size and location with known coordinates of the deletion in the literature and our cases. Black bars: Critical regions proposed for specific phenotypic features of 5p-. Gray bars: The deletion of previously reported 5p- cases with detailed breakpoint. Red bars: our six pure 5p- cases. EF: epicanthal folds, GD: growth deficiency, FD: facial dysmorphology, SD: speech delay, ID: intellectual disability, CHD: congenital heart disease, RA: renal anomalies, BM: brain malformations, VM: ventriculomegaly, ISS: increased subarachnoid space, VH: vermis hypoplasia, SUA: single umbilical artery, CSPD: cavum septum pellucidum dilation, CH: cerebellar hypoplasia, DCV: dysgenesis of the cerebellar vermis, CPC: Choroid plexus cyst, Iβ-HCG: increased β-HCG, CML: cystic adenomatoid malformation of the lung, INF: increased NF

In total, 12 prenatal cases with 5p terminal deletion were detected in our cohort by SNP array, the median maternal age was 29 (21–43) years and the median gestational age at prenatal diagnosis was 20.5 (13–29) weeks. Full descriptions of these cases were list in Table 1. Cases 1–6 with pure 5p terminal deletion, case 7–12 with additional pathogenic chromosome abnormalities, two cases (case 7, 10) were referred to the hospital at the first trimester because of an increased NT, the indication of case 1 and case 12 were abnormal NIPT finding for high risk of 5p- and trisomy 18, respectively. In total, 10 cases were identified as 5p- syndrome due to abnormal ultrasound findings at second trimester. Karyotyping results were available of nine cases (case 1–6, 10–12), all the parents were determined to terminate the pregnancy by considering the poor prognosis, only three cases (case 5, 6, 10) preformed parental chromosome analysis. Detailed size and the coordinates of the pure 5p terminal deletions were shown in Fig. 1. Table 2 summarized the ultrasound signs of the prenatal CdCS cases with pure 5p terminal deletion from the literatures including the twelve cases in our cohort.

**Table 1:** Detailed clinical information of our twelve cases with 5p- syndrome

Index	Maternal age (years)	Gestation (w + d)	G-band	Snp array results	Ultrasound finding	Inheritance
1	26	23 + 1	46,XX,del(5)(p13)	arr [hg19] 5p15.33p13.3 (38,139-30,536,972)× 1	(NIPT at 13 weeks: high risk for 5p-), Cerebellar hypoplasia	
2	27	24 + 2	46,XY,del(5)(p14)	arr [hg19] 5p15.33p14.3 (464,153-23,132,422)×1	Dysgenesis of the cerebellar vermis	
3	43	16 + 6	46,XY,del(5)(p15)	arr [hg19] 5p15.33p15.1 (38,139-17,981,307)× 1	Choroid plexus cyst	
4	26	22	46,XX,del(5)(p15)	arr [hg19] 5p15.33p15.31 (38,139-9,782,775)×1	Abnormal maternal serum screening (increased β-HCG)	
5	30	22 + 6	46,XX,del(5)(p14)	arr [hg19] 5p15.33p14.3 (38,139-19,508,190)× 1	Cystic adenomatoid malformation of the lung	de novo
6	31	21 + 1	46,XX,del(5)(p14)	arr [hg19] 5p14.3p15.33 (1,151,161-20,687,905)× 1	NF:6.8 mm	De novo
7	35	13 + 2	NA	arr [hg19] 5p15.33p14.3 (38,139-22,612,407)×1,13q31.2q34 (89,736,965-115,106,996)×3	Hydrops fetalis, NT:8.7 mm	
8	27	29 + 0	NA	arr [hg19] 5p15.33p15.1 (354,051-17,484,038)×1,5q34q35.3 (165,731,079-180,705,539)×3	VSD, NF:9 mm, abnormality of lateral ventricle	
9	37	23 + 5	NA	arr [hg19] 5p15.33p15.1 (560,476-17,910,453)×1, 8q24.12q24.3(119,864,143-146,293,086)× 3	Cerebellar hypoplasia	
10	21	14 + 5	46,XX, der(5)t(5;18)(p15.2;q11.2)	arr [hg19] 5p15.33p15.2 (38,139-10,702,034)× 1,18q11.2q23(19,178,726-78,014,582)× 3	NT:5.5 mm, abnormal heart valve morphology, absent of the nasal bone	paternal 46,XY, t(5,18)(p15.1,q11.2)
11	27	18	46,XY,der(5)t(5;7)(p15;q21)	arr [hg19] 5p15.33p15.2 (38,139-12,392,815)× 1, 7q21.11q36.3 (83,599,335-159,119,486)× 3.	SUA, DLV, talipes equinovarus, abnormality of the cerebral ventricles	
12	26	21 + 2	46,XX,der(5)t(5,18)(p15.2,q12.3)	arr [hg19] 5p15.33p15.2 (560,476-14,855,659)×1, 18q12.3q23(43,354,452-78,014,582)×3.	CLP, VSD, NF:6.5 mm, (NIPT: high risk for T18)	

*w* Weeks, *d* Days, *NA* Not analysis, *NT/NF* Nuchal translucency/nuchal fold thickness, *VSD* Ventricular septal defect, *SUA* Single umbilical artery, *DLV* Dilation of lateral ventricles, *CLP* Cleft lip and palate, *β-HCG* Beta-human chorionic gonadotropin.

**Table 2:** Summary of the CdCS cases with pure terminal 5p deletion from the literature compared to the 6 cases in our cohort^a^

Prenatal ultrasound signs	Previously reported(*n* = 30)	Our group(*n* = 6))	Total
Increased NT/NF	2/30	1/6	3/36
IUGR	1/30	0/6	1/36
Aplasia/Hypoplasia of the cerebellum	7/30	2/6	9/36
Abnormality of the cerebral ventricles	7/30	0/6	7/36
Absent/severely hypoplastic nasal bone	2/30	0/6	2/36
Choroid plexus cyst	4/30	1/6	5/36
Single umbilical artery	4/30	0/6	4/36
VSD	3/30	0/6	3/36
Hydrops fetalis	3/30	0/6	3/36
Ascites	3/30	0/6	3/36
Encephalocele	1/30	0/6	1/36
Others			
Abnormal serum markers (β-hCG, PAPP-A)	7/30	1/6	8/36
AMA without abnormal US finding	3/30	0/6	3/36

-^a^Data collected from the 30 prenatal cases with pure distal 5p deletion from 24 studies (Additional file 1: Table S1). *IUGR* Intrauterine growth retardation, *VSD* Ventricular septal defect, *AMA* Advanced maternal age, *US* Ultrasound.

## Discussion and conclusion

In the current study, we provided a largest series in prenatal CdCS cases detected by SNP array in China. The incidence of CdCS in our cohort was around 0.034% (12/35,233), much higher than the previously reported incidence of 1/50,000–1/15,000 in live births [25]. According to our retrospective analysis, 6/12(50%) of our cases had an isolated 5p terminal deletion, the proportion (54.5%) was lower than that (62.5%) reported by Han et al. in eight prenatal cases [20] and greater than that (20%) reported by L. Mark et al. [22]. 6/12(50%) of cases characterized by unbalanced translocation, the sex ratio of our CdCS cases was 7/5 (female/male), consistent with the rate of postnatal series by Nguyen, J. M.et al. [2].

Prenatal CdCS cases were difficult to be found in first trimester screening (FTS). Less than 50 isolate 5p deletion prenatal cases had been reported since Lejeune et al. described the first 5p- syndrome cases in 1963 [1]. Though abnormal level of serum human chorionic gonadotrophin (β-HCG) as well as PAPP-A presented in some of CdCS cases (22.2%, 8/36) similar to our case 4 [10], the association between the abnormal FTS markers and CdCS were yet to be established. NIPT has rapidly become a part of the standard prenatal care for aneuploidy screening for high-risk pregnancies in our region. Case 1 was screening with CdCS using NIPT with high risk for a large chromosome 5p deletion at 13 weeks. At 23 weeks, the fetus presented cerebellar hypoplasia and amniocentesis SNP array revealed a 29.8 Mb deletion of 5p15.33p13.3. Case 12 indicated a high risk for trisomy 18 by NIPT, fetus ultrasound feature included cleft lip and palate, ventricular septal defect, and increased NF were found at 21 weeks, consistent with the features of trisomy 18. SNP array revealed a 35 Mb duplication at 18q12.3q23 and a 9.8 Mb deletion at 5p15.33p15.2. We believe that NIPT method would be one very promising screening tool for CdCS in FTS [26], however, due to the low prevalence of CdCS in the population, clinical application of NIPT on CdCS was limited by a low positive predictive values [26–28] .

Abnormal prenatal findings associated with pure 5p- syndrome were summarized in Table 2. Collectively, 13 cases (16/36, 44.4%) were described involving the cerebral abnormalities. Other recurrent phenotypes included: Choroid plexus cyst in 5 patients (13.8%), Single umbilical artery in 4 patients (11.1%), ventricular septal defect in 3 cases (8.3%), hydrops fetalis in 3 cases (8.3%), ascites in 3 cases (8.3%), increased NT/NF in 3 cases (8.3%), absent/severely hypoplastic nasal bone in 2 cases (5.5%), abnormal serum markers (hCG, PAPP-A) in 6 cases (22.2%). Three cases carried out chromosomal analysis for advanced maternal age with 5p- without abnormal US finding, one case with IUGR and one case with encephalocele. Simmons et al. proposed that deletion of 5p15.2 was responsible for abnormal cerebral development [29], in our series, 41.6% (5/12) of cases involving 5p15.2 include two pure 5p- cases (case 1, 2) and three unbalanced translocation (case 8, 9, 11) were found with cerebellar abnormalities, these data presented here indicated that cerebral abnormalities could probably become the most frequent prenatal feature of CdCS.

In previous prenatal studies, most of CdCS cases were diagnosed by routine G-banding karyotyping, which are observable only when the deletion size was larger than 5 Mb at the resolution approximately 400 bands. Eleven pure 5p- cases reported here with detail breakpoint without consistent features were observed (Fig.1). Multiple critical regions for the clinical feature were proposed, a 640 kb region at 5p13 and a 1.7 Mb localized the 5p15.31p15.32 region were characteristic for cat-like cry [30, 31]. Regions related with speech delay, mental retardation had been proposed as 3.2 Mb in 5p15.32–15.33, 5p15.2p15.1 with a 5.4 Mb~ 5.5 Mb [7, 32] and a 2.4 Mb in 5p15.2–15.31 [31], respectively. Other facial dysmorphology features such as microcephaly, micrognathia, hypertelorism were reported in the 5.5 Mb region by Kondoh et al. [32]. These critical region sizes were too small and beyond the G-band’s resolution. Furthermore, some of 5p- syndrome cases might also be missed by conventional cytogenetic analysis alone [22]. Thus, combined CMA with karyotyping could be a definitive method for diagnosis of 5p- syndrome, and it can be expected that more cases of CdCS would be detected since routine clinical use of CMA in prenatal.

Determining the mechanism leading to the 5p- is essential for counseling and the recurrence risk estimation. Most of 5p deletions were commonly de novo occurrences, about 80–90% of CdCS cases were results from terminal deletions, approximately 15% of CdCS had an unbalanced translocation, de novo or inherited. Other mechanisms include interstitial deletions (3–5%), mosaicism (1.4%), inversions (0.5%), or ring chromosomes (0.5%) [5, 8]. The molecular and cytogenetic tests of prenatal diagnosis in our state remains entirely patients-funded, most of pregnant women with the 5p deletion decided to termination of pregnancy directly without perform any further molecular tests based on consideration of the poor prognosis, we had no further information to evaluate the inheritance in these cases. This highlights the importance of genetic counseling for the families with high risk of translocation involving 5p deletion, for the risk of producing a CdCS off-spring was estimated approximately 8.7 to 18.8% [4].

In summary, we presented the first largest prenatal series of CdCS diagnosed by SNP array in China. Though cerebellar abnormalities could be the most frequent features in prenatal CdCS cases, the prenatal ultrasound features of CdCS remain non-specific. When cases with ultrasound findings such as cerebellar abnormalities, absent/severely hypoplastic nasal bone, choroid plexus cyst, single umbilical artery, ventricular septal defect, hydrops fetalis, ascites, encephalocele are observed, a clinical suspicion of CdCS or other microdeletion/duplication syndromes should be considered. Combining typical karyotyping with CMA is a definitive method for a precise diagnosis of CdCS. Our findings expand the prenatal phenotypes of CdCS and highlight the importance of prenatal ultrasonography for the detection of chromosomal aberrations.

## Additional file

**Additional file 1: Table S1.** Thirty-six prenatal cases with pure 5p terminal deletions were reviewed from 24 published articles included our cases.

## Data Availability

Please contact authors for data requests.

## Abbreviations

AMA: Advanced maternal age
CdCS: Cri-du-chat syndrome
CLP: Cleft lip and palate
FTS: First trimester screening
IUGR: Intrauterine growth retardation
NIPT: Non-invasive prenatal testing
SNP array: Single nucleotide polymorphism array
US: Ultrasound
VSD: Ventricular septal defect
β-HCG: Beta-human chorionic gonadotropin
