# Supplementary material for: Prenatal diagnosis of cri-du-chat syndrome by SNP array: report of twelve cases and review of the literature
Source: Mol Cytogenet. 2019 Dec 9;12:49. doi: 10.1186/s13039-019-0462-0 (PMC6902614; doi:10.1186/s13039-019-0462-0)
Supplement: Supplementary file 1 — Additional file 1: Table S1. Thirty-six prenatal cases with pure 5p terminal deletions were reviewed from 24 published articles included our cases. [file 13039_2019_462_MOESM1_ESM.docx]

Table S1. Thirty-six prenatal cases with pure 5p terminal deletions were reviewed from 24 published articles included our cases.

| **pure 5p- cases** | **cytogenetic result** | **molecular genetic result(GRCh37/hg19)** | **Ultrasound and screening test findings** |
| --- | --- | --- | --- |
| Mak et al., 2019 | 46,XX,del(5)(p15.1) | arr5p15.33p15.1(55550-17114507)x1 dn | Fetal cardiomegaly, pericardial effusion, reduced transverse cerebellar diameter and prominent lateral ventricles. |
| Cardoso et al., 2019 | 46,XX,del(5) (p15.2) |  | Isolated fetal ascites |
| Kaymak et al., 2019 |  | arr 5p15.33p14.3 (22,149–217,617,93)x1 dn | Bilateral mild ventriculomegaly, increased subarachnoid space, pontocerebellar and vermis hypoplasia |
| Vado et al., 2018 | 46,XX,del(5)(p15.2) | arr5p15.33p15.2(25942–11644643)× 1 | Single umbilical artery |
| Brabbing-Goldstein et al., 2018 |  | arr 5p15.33p13.3 (1-29,521,914)x1 | Cerebellar hypoplasia |
| Han and Kwak 2017 | 46,XX,del(5)(p13) |  | Fetal hydrops was diagnosed with multiple congenital anomalies, including increased nuchal translucency (INT), hydrops of the fetus, and an abnormal heart axis at the first |
|  | 46,XX,del(5)(p13.3) |  | Small-sized cerebellum, unilateral ventriculomegaly |
|  | 46,XX,del(5)(p13.3) |  | INT (2.8 mm) and mild soft tissue edema of the fetus |
|  | 46,XY,del(5)(p14). |  | AMA:advanced maternal age US: normal |
|  | 46,XY,del(5)(14). |  | AMA:advanced maternal age US: normal |
| Gonzalez-Comadran et al., 2015 | (46XY,?del(5)(p13)) | arr5p15.33p14.1(131,946–26,885,727)x1, | Unilateral renal dysplasia, a mild dilation of the cavum septum pellucidum |
| Macayran Nguyen et al., 2014 | 46, XX, del(5)(p15.2) | 9.837- to 9.924-Mb deletion at 5p15.33p15.2. | Single-umbilical artery, |
| Xu et al., 2014 | 46,XX | arr 5p15.33(711,476-1,411,321)x1 | normal |
| Chen et al., 2013 | 46, XX, del(5)(p15.1) | arr 5p15.33p15.1 (679,395– 15,844,407) × 1. | Cerebellar hypoplasia and hypospadias |
| Li and Yi 2012 | 46,XX,del(5)(p15) |  | VSD, Horseshoe kidney, SUA |
|  | 46,XX,del(5)(p15) |  | VSD, Overriding aorta |
|  | 46,XX,del(5)(p15) |  | Bilateral ventriculomegaly |
|  | 46XX,del(5)(p14) |  | Maternal inv(7)(p23;q32) no US sings |
| Li 2009 | 46,XY,del (5) (p14) |  | Normal ultrasonographic ﬁndings with increased β-hCG |
| Torun et al., 2009 | 46, XY, del (5p) (pter → p13) |  | bilateral choroid plexus cysts, increased β-hCG |
| Teoh et al., 2009 | 46,XX,del(5)p14 |  | Hypoplastic nasal bone, hypoplastic cerebellum, right -sided choroid plexus cyst, and a single umbilical artery. no thickened nuchal fold |
| Sherer et al., 2006 | 46,XX,del(5)(p15.1) |  | absent nasal bone |
| Vialard et al., 2005 |  | 46,XY,del(5p12),del ish | Dandy–Walker malformation with a large dilatation and protrusion of the fourth ventricule and vermis agenesia. Agenesis of the corpus callosum with absence of the pericallosal artery and a radial aspect of the vascularisation. Abnormal proﬁle with a ﬂat nose and microretrognathia |
| Bakkum et al., 2005 | 46,XY,del(5)(p13) |  | posterior cranial abnormality consistent with an encephalocele |
| Chen et al., 2004 | 46,XX,del(5)(p15.1)[23]/46,XX[23] |  | microcephaly, cerebellar hypoplasia |
| Stefanou et al., 2002 | 46,XY, del(5)(p15) |  | Theonly abnormality that could be seen was moderate bilateral cerebral ventriculomegaly with the anterior and posterior horn of lateral ventricle measuring 10.4 mm and 10.6 mm, respectively, mild ventriculomegaly |
| Aoki et al., 1999 | No detail except 5p- |  | VSD, pleural effusion, ascites, skin edema, a hypoplastic cerebellum, Hydrops fetalis |
| Fankhauser et al., 1998 | 46,XN,del(5)(p15.1) |  | fetal choroid plexus cyst had beennoted by ultrasonography at 18 weeks, especially no growth retardation |
| Tullu et al., 1998 | 46,XY,del(5)(p15). |  | Hydrops fetalis ,fetal ascites and bilateral hydronephrosis |
| Sarno et al., 1993 | 46,XX,del(5)(p13) |  | Fetal choroid plexus cysts ,IUGR |
| David et al., 1978 | 46, XY, 5p-. |  | AMA:advanced maternal age US: normal |
| Our case 1 | 46,XX,del(5)(p13) | arr5p15.33p13.3(38,139-30,536,972)x1 | Choroid plexus cyst |
| case 2 | 46,XY,del(5)(p14) | arr5p15.33p14.3 (464,153-23,132,422)x1 | Abnormal maternal serum screening (increased β-HCG:4.98) |
| Case 3 | 46,XY,del(5)(p15) | arr5p15.33p15.1(38,139-17,981,307)x1 | Cystic adenomatoid malformation of the lung |
| Case 4 | 46,XX,del(5)(p15) | arr5p15.33p15.31(38,139-9,782,775)x1 | NF:6.8mm |
| Case 5 | 46,XX,del(5)(p14) | arr5p15.33p14.3(38,139-19,508,190)x1 | Cystic adenomatoid malformation of the lung |
| Case 6 | 46,XX,del(5)(p14) | arr5p14.3p15.33(1,151,161-20,687,905)x1 | NF:6.8mm |
